# PM_2.5_ Prediction with a Novel Multi-Step-Ahead Forecasting Model Based on Dynamic Wind Field Distance

**DOI:** 10.3390/ijerph16224482

**Published:** 2019-11-14

**Authors:** Mei Yang, Hong Fan, Kang Zhao

**Affiliations:** State Key Laboratory of Information Engineering in Surveying, Mapping and Remote Sensing, Wuhan University, 129 Luoyu Road, Wuhan 430079, China; yangmei2012@whu.edu.cn (M.Y.); kzhao@whu.edu.cn (K.Z.)

**Keywords:** PM_2.5_ prediction, spatiotemporal correlation, long short-term memory neural network, convolutional neural work, KNN

## Abstract

Particulate matter with a diameter of less than 2.5 μm (PM_2.5_) has damaged public health globally for a decade. Accurate forecasts of PM_2.5_ concentration can provide early warnings to prevent the public from hazard exposure. However, existing methods have not considered the available spatiotemporal data sufficiently due to their architecture or inadequate input, and most neglected wind impact on spatiotemporal correlation when selecting related sites. To fill this gap, we proposed a long short-term memory-convolutional neural network based on dynamic wind field distance (LSTM-CNN-DWFD) to predict PM_2.5_ concentration of a specific site for the next 24 h. A KNN method based on dynamic wind field distance was developed and applied to select highly related sites considering wind impact. A local stateful LSTM model was employed to capture temporal correlations in historical air quality and meteorological data for each related site. Then, these temporal features were integrated as a spatiotemporal matrix, and input into CNN for extracting spatiotemporal correlation features. Weather forecasts were also integrated into the model to promote accuracy. Hourly PM_2.5_ data from 36 monitoring sites in Beijing, China collected from 1 May 2014 to 30 April 2015 were used as experimental dataset. Six-fold rolling origin method was employed to conduct experiments on each site, and the results of 216 experiments validated the performance of the proposed LSTM-CNN-DWFD model. The mean $R^{2}$ values of the next 1–6 h prediction were 0.85, 0.81, 0.76, 0.70, 0.64, and 0.59, respectively, showing a decrease trend, indicating that the prediction accuracy decreases as the prediction time increases. Comparisons of LSTM-CNN-DWFD results to results from six other methods show that it delivered higher accuracy PM_2.5_ predictions, with the mean RMSE (MAE) of 1–6, 7–12, and 13–24 h prediction were 43.90 (29.17), 57.89 (42.16), and 63.14 (47.64), respectively. The results also demonstrate that the sites selected based on dynamic wind field distance are more related to the central site than that based on geographical distance, also contributing to prediction accuracy.

## 1. Introduction

Over the past decades, the serious pollution problems caused by the rapid global economic development have attracted the attention of both researchers and the general public worldwide. According to the World Health Organization [1], approximately 90% of people worldwide breathe air that does not accord with air quality standards, and around three million deaths each year are related to outdoor air pollution. Particles suspended in the air can harm the respiratory and cardiovascular systems of the human body, possibly leading to cardiopulmonary diseases [2,3]. The concentration of PM_2.5_ reflects the degree of contamination. The full recognition and accurate forecasting of PM_2.5_ concentration can guide the government in taking timely actions to reduce the pollution emission and prevent hazard exposure for the public; thus, related methods have undergone extensive studies [4,5,6,7].

Methods for predicting PM_2.5_ concentrations can be divided into two types, empirical and physical models. Physical models adopt meteorological principles and statistical methods to simulate the emission, dispersion, transformation, diffusion, and removal processes of pollutants [7,8]; thus, the spatiotemporal distribution of air pollutants are predicted. Physical models can provide explicit insights into the physical-chemical processes of the diffusion and transformation of multiple pollutants and present the direct linkage between pollutant emission and air pollution. However, the physical models are dependent on priori knowledge and accuracy of emission data, which may cause errors. Comparative analyses have demonstrated that well-developed site-specific empirical models performed higher prediction accuracy than physical methods [9]; thus, we explored an empirical model to predict air quality in this study. Empirical studies avoid complicated theoretical models; they simply adopt statistics-based methods to predict the air quality, and many empirical models have been proposed. The air quality and weather of surrounding sites affect the PM_2.5_ concentration of the central site due to the diffusion of pollutants and other meteorological elements across areas. This condition refers to spatial correlation. Traditional empirical methods address spatial correlation by two means. One is to consider all sites in the study area [10,11]. However, sites farther away from the target site can reduce the prediction accuracy of the models. In addition, these methods are hard to handle prediction tasks across a large area. The other only takes the K-nearest neighbor (KNN) sites into consideration, and it has been extensively applied in PM_2.5_ forecasting for selecting the most related sites [12,13,14]. In these studies, the distance between the target site and other sites were calculated, and the K-nearest sites were selected and input to the model for training. Thus, it can decrease the number of input features, and avoid the interference of less relevant sites. The most popular distance criterion employed is the geographical distance, but pollutant diffusion processes are based on wind. However, these models neglected wind when selecting related sites.

Researchers have extensively used neural network methods to predict air quality [15,16,17,18,19]. Most of these methods are based on multivariate linear model and multilayer perception, which are not designed for time series data (e.g., PM_2.5_ concentration). They performed lower accuracy than recurrent neural networks, such as long short-term memory (LSTM) neural network, in handling temporal correlation [20]. Compared with ordinary recurrent neural networks, LSTM [21] inherently captures long- and short-term trends and effectively avoids exploding and vanishing gradient problems; thus, it is widely used in time series forecasting [13]. Li and Peng [11] incorporated the historical PM_2.5_ concentration of 12 monitoring sites in Beijing with their meteorological data into the LSTM model and simultaneously provided predictions of hourly PM_2.5_ concentration of all sites. Soh [14] and Zhao [22] employed LSTM and fully connected layer (FC) neural network to extract spatiotemporal correlation features from the historical air quality and meteorological data of target and neighbor sites. They found that LSTM has higher prediction accuracy than other models, such as ordinary recurrent neural network and support vector regression model. However, the spatial correlation was not fully considered in the above studies. The employed FC is designed for processing 1D characteristic. Therefore, high-dimensional spatial features can only be input into FC after flattening, possibly causing loss of spatial information. Comparatively, convolutional neural network (CNN) [23] extracts spatial correlation features by convolving the adjacent elements of high-dimensional matrices with moving kernels. Consequently, it can fully use spatial information. CNN has been applied widely in the computer vision field to capture the spatial features of images [24,25]. When the data of multiple related sites are integrated into a matrix, then, its elements are correlated due to spatial correlation. Thus, CNN is suitable for extracting spatial features of multiple sites. Qin and Yu [26] proposed a two-step forecasting model that combined CNN to capture the spatial features of air quality and meteorological data of multiple sites and LSTM to learn the temporal correlations. However, instead of predicting the PM_2.5_ concentration of a specific site, this model aimed to make predictions of the target city, which cannot provide the spatial distribution of pollution. Huang and Kuo [27] proposed APNet model that used CNN to capture the correlation among the air quality and meteorological data of the target station and LSTM to extract temporal features. Wen and Liu [28] used CNN to capture the spatial correlation of the PM_2.5_ data of multiple stations and then used LSTM to capture temporal tendency. However, both of them have not considered the air quality and meteorological spatiotemporal data of surrounding sites sufficiently.

The current study proposed an LSTM-CNN multi-step-ahead forecasting model using dynamic wind field distance (LSTM-CNN-DWFD) to fill the gap of existing methods. First, we selected K-nearest sites as neighbor sites in accordance with defined dynamic wind field distance instead of the most popular geographical distance. Second, we built an LSTM-CNN hybrid neural network to provide predictions. We input the historical air quality and meteorological condition of the target site and neighbor sites into a local LSTM and FC to capture temporal features of each site. Then, we used CNN to capture spatiotemporal features. We also concatenated the spatiotemporal features with the weather forecasts. In turn, we input these forecasts into FC to predict the hourly PM_2.5_ concentration of the target site over the next 24 h. We selected the PM_2.5_ and weather data of Beijing collected from 1 May 2014 to 30 April 2015 as experimental data, and conducted six-fold rolling origin experiments on 36 target monitoring stations. The comparison results with other six methods confirmed the effectiveness and superiority of the proposed model in predicting PM_2.5_ concentration. This study makes the following contributions: (1) When using KNN to select related sites, wind impact was taken into consideration by replacing geographical distance with dynamic wind field distance; (2) spatiotemporal data, which includes the air quality and weather data of the target and neighbor sites, were fully considered in the proposed LSTM-CNN-DWFD model. From the results, the proposed method was found more effective in selecting related sites and extracting spatiotemporal features and performed with higher accuracy in multi-scale predictions of PM_2.5_ concentration than other methods.

## 2. Materials and Methods

**Figure 1:**
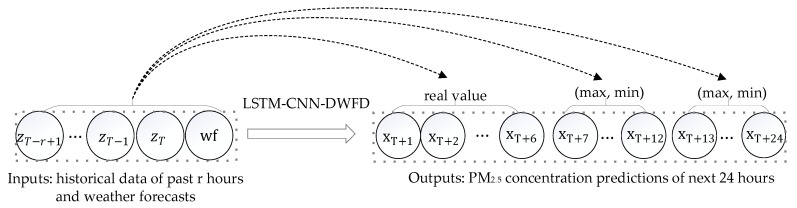
Multi-step-ahead forecasting model. r refers to the time lag; $z_{T-r+1}$ to $z_{T}$ refer to the historical data at time T−r+1 to T, including air pollution and meteorological factors; wf refers to weather forecasts of the target site; $x_{T+1}$ to $x_{T+24}$ refer to PM_2.5_ concentration at time T+1 to T+24.

This study aims to forecast a specific site’s hourly PM_2.5_ concentration of the next 24 h. We divided 1–24 h into three intervals, that is, 1–6, 7–12, and 13–24 h, and trained three LSTM-CNN-DWFD forecasting models for them. As shown in Figure 1, the historical data of past r hours were input into the model. We forecasted the real-valued concentration of PM_2.5_ for the next 1–6 h and the maximum and minimum values of the next 7–12 and 13–24 h. Therefore, the proposed model is a multi-step-ahead forecasting model. In the following subsections, we introduce the details of the proposed LSTM-CNN-DWFD model, the experimental datasets, and the evaluating methods.

### 2.1. Data

**Figure 2:**
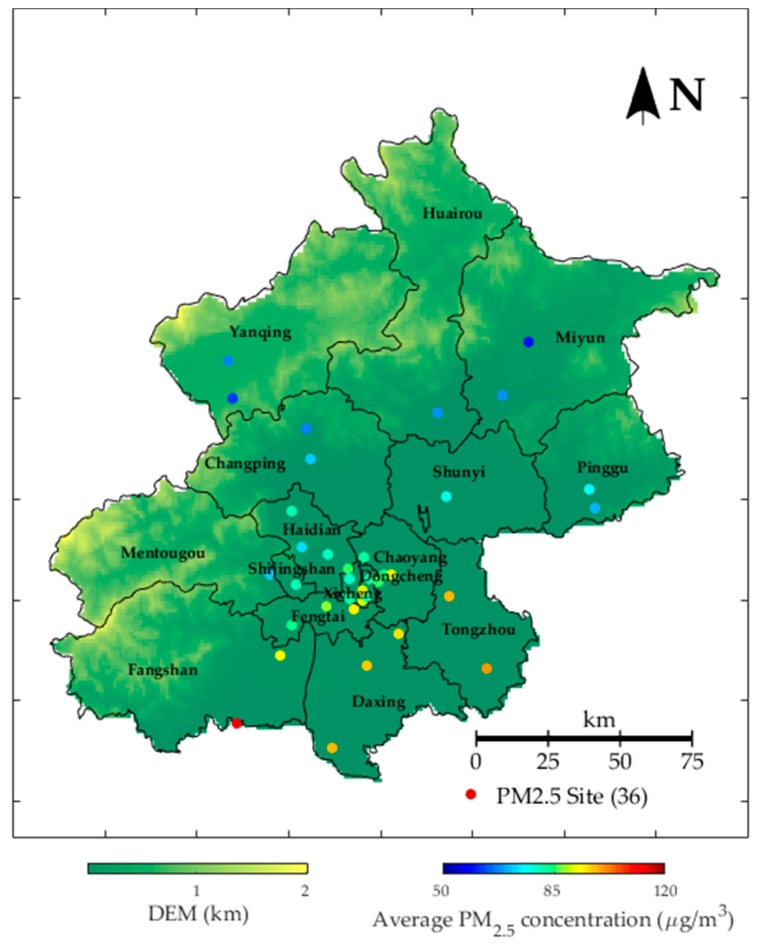
Locations of air quality stations in Beijing. The color represents the rank of the average PM_2.5_ concentration during 1 May 2014 to 30 April 2015 as described in the bottom right of the figure.

As the capital of China, Beijing is one of the most economically dynamic cities and the most polluted cities. According to the dataset collected from Urban Air project of Microsoft Research [29], the air quality and meteorological data measured in Beijing was selected as the experimental data. This project was established for using a diversity of big data to infer and predict fine-grained air quality throughout a city, and finally tackle air pollutions [10,30,31]. This project collected real-time meteorological data, weather forecasts, and air quality data of different cities from a list of public websites and public/private web services. Hourly air quality data from 2296 stations in 302 Chinese cities were collected, including PM_2.5_, PM_10_, NO_2_, CO, SO_2_, and O_3_. The meteorological data were collected from 3514 cities/districts/stations, which included observations of time, weather, temperature, pressure, humidity, wind speed, and wind direction. Additionally, weather forecasts data were also collected from 2612 cities/districts, including weather, wind direction, wind level, and up and down temperature. The whole dataset of four major Chinese cities (Beijing, Tianjin, Guangzhou, and Shenzhen) were organized as general text files and open to the public for further researches [32], and more details of this dataset can be found in Zheng [10]. This dataset has been applied to many recent empirical researches for exploring PM_2.5_ concentration forecasting models [12,14,22,33,34]. According to this dataset, we selected the hourly data related to Beijing as experimental data, including the air quality records of 36 monitoring stations, meteorological observations of meteorological stations, and weather forecasts. Its time period is from 1 May 2014 to 30 April 2015. Figure 2 shows the locations of the 36 monitoring stations. The color represents the grading of the average PM_2.5_ concentration. Discrete variables, namely, wind level, wind direction, and weather, were input into the embedding layer to reduce the dimensionality.

The air quality and meteorological observations contained some missing values. We filled these missing values via two means: If the values of the previous hour and the subsequent hour existed, then, we interpolated the missing value via linear interpolation based on these values; otherwise, we filled the missing value via inverse distance interpolation in accordance with the values of other sites. The interpolation accuracy was tested by randomly interpolating 100 valid PM_2.5_ concentration observations. The mean absolute error (defined in Section 2.3) was 7.85 $\mu g/m^{3}$. Finally, all observations were centralized and standardized by the following equation to eliminate the impact of different dimensions.

(1) $$x_{i}^{*}=\frac{x_{i}-\bar{x}}{\sigma }$$

where $x_{i}$ and $x_{i}^{*}$ represent the original observation and transformed values of factor x, respectively; $\bar{x}$ and σ are the mean and standard deviation of all observations, respectively.

### 2.2. Methods

**Figure 3:**
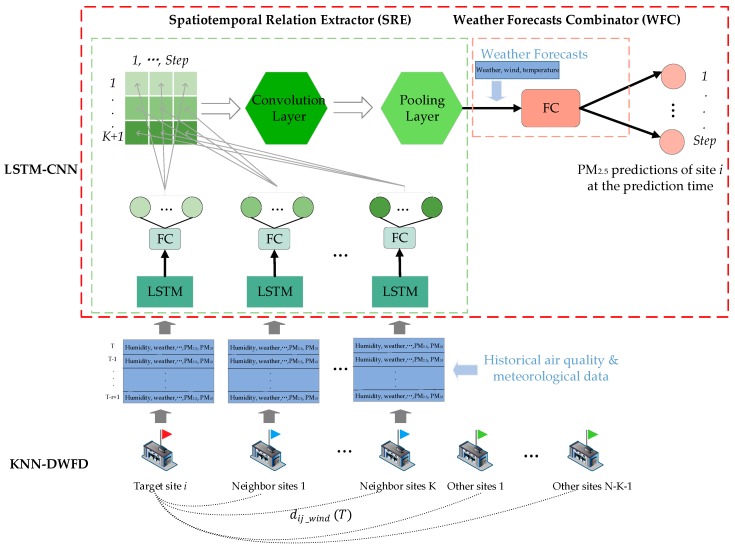
Overall framework of the proposed long short-term memory-convolutional neural network based on dynamic wind field distance (LSTM-CNN-DWFD). The red, blue, and green flags at the bottom of the figure stand for the target site, K-nearest neighbor sites, and other sites, respectively. Step means the dimension of forecast; it equals to 6 for 1–6 h forecasting, whereas it equals to 2 for 7–12 and 13–24 h forecasting.

Figure 3 presents the overall framework of the proposed LSTM-CNN-DWFD. The input (see the blue arrows in Figure 3) originated from two parts: The historical air quality and meteorological data of the target and neighbor sites at past r hours and weather forecasts of the target station. The output was the PM_2.5_ concentration predictions of the target station at T+1,…,T+24. The architecture of LSTM-CNN-DWFD consisted of two steps: Selecting highly related sites using KNN based on dynamic wind field distance (KNN-DWFD) and extracting spatiotemporal features using a hybrid neural network LSTM-CNN. Particularly, LSTM-CNN contained a spatiotemporal relation extractor (SRE) and a weather forecast combinator (WFC) as shown in Figure 3. The processes of KNN-DWFD and LSTM-CNN are as follows.

*KNN-DWFD*. As shown at the bottom of Figure 3, the red flag represents the target station to be predicted. We defined dynamic wind field distance to evaluate the distance between the target and other sites at time T. Then, we selected the K-nearest stations as the most related sites, and we defined them as neighbor sites to the target site (blue flags in Figure 3). The historical air quality (PM_2.5_, PM_10_, NO_2_, CO, SO_2,_ and O_3_) and meteorological data (weather, temperature, pressure, humidity, wind speed, and wind direction) of the target and neighbor stations at past r hours (T−r+1,…,T) were input into the following model. The wind impact on spatial correlation was fully considered, and the interference of less relevant sites was avoided by the selection process.

*LSTM-CNN: SRE*. As the green part in Figure 3 shows, we constructed an SRE to extract spatiotemporal features from the above historical spatiotemporal data. Local LSTM was built on each site to learn the long- and short-term tendencies of historical data. By decoding the outputs of FC, the temporal features were obtained for each site. Finally, we employed CNN to learn spatiotemporal correlation among these temporal features. The compressed spatiotemporal features were output by the convolution and pooling layers.

*LSTM-CNN: WFC*. The weather forecasts (i.e., weather, wind direction, wind level, and up and down temperature) of the target station were integrated with the spatiotemporal features obtained by SRE into an FC layer (see the pink part in Figure 3), and the final predictions of the future PM_2.5_ concentration of target station were output.

The three parts were combined together to form LSTM-CNN-DWFD. The details of each part are introduced in the following subsubsections.

#### 2.2.1. KNN-DWFD

##### Traditional KNN and Its Limitations

KNN [35] aims to calculate the distance between a sample and other samples in accordance with their characteristics and to choose the

**Figure 4:**
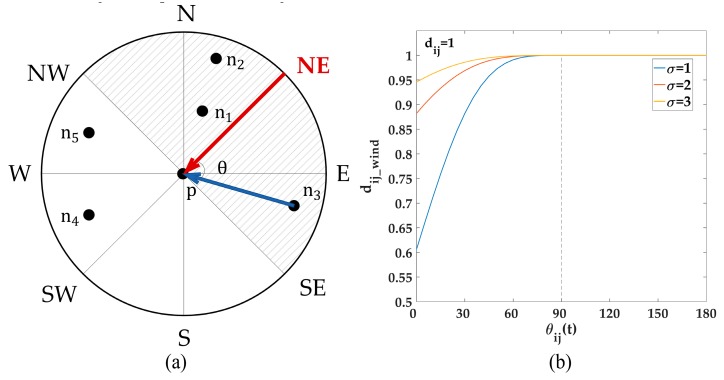
(**a**) Illustration of the spatial correlation of PM_2.5_ concentration. (**b**) Variation curve between $d_{ij\_wind}\left(t\right)$ and $\theta_{ij}\left(t\right)$ when σ=1,2,3 and $d_{\mathrm{ij}}=1$.

K-nearest samples as the most related ones. In this paper, we define the K most related sites as neighbor sites to the target site. KNN has been extensively employed to choose the most related sites in the pollution field due to its high computation efficiency. The most commonly used distance criterion is the geographical distance, and the formula is as follows:

(2) $$d_{ij}=\sqrt{{\left(x_{i}-x_{j}\right)}^{\prime }\left(x_{i}-x_{j}\right)}$$

where $x_{i}$ and $x_{j}$ are the locations (longitude, latitude) of stations i and j, respectively, and $d_{ij}$ represents their geographical distance. This distance considers spatial correlation to be related only with geographical distance. Therefore, the spatial correlation is considered to be isotropic by this definition of distance. However, the diffusion of pollutants is based on wind direction and wind speed. As such, the spatial correlation should be considered to be anisotropic. For example, in Figure 4a, p is the target station and its wind direction is northeast (the red arrow in Figure 4a); the upwind region is denoted as shadow area. $n_{1},\;n_{2},\;\ldots ,\;n_{5}$ are the surrounding monitoring stations that locate at different directions. Evidently, $n_{1},\;n_{2},$ and $n_{3}$ are at the upwind area, and their pollutants are transported to p depending on the wind. Therefore, their affect degree to p are higher than that indicated by the geographical distance. Moreover, the nearer to the target station and the closer to the upwind direction, the easier the pollutants can be transported to p, thus the higher the affect degree of the surrounding sites. We define $\theta_{ij}\left(t\right)$ to represent the angle between the wind direction of target site i at time t and the edge between i and a surrounding site j (the blue arrow in Figure 4a), and $d_{ij}$ represents the geographical distance between sites i and j. Then, owing to the transport process based on wind, the spatial correlation is negative with $d_{ij}$ and $\theta_{ij}\left(t\right)$. Moreover, the affect degree of upwind sites is higher than that of downwind sites with the same distance.

##### Definition of KNN-DWFD

The traditional geographical distance $d_{ij}$ can only represent the dependence of spatial distance; it cannot describe the effect of wind direction. To address the negative correlation between spatial relation with $d_{ij}$ and $\theta_{ij}\left(t\right)$ and simultaneously increase the affect degree of upwind sites, a dynamic wind field distance based on Gauss kernel function $d_{ij_{wind}\;}\left(t\right)$ was presented as shown in the following equation.

(3) $$d_{ij\_wind}\left(t\right)=\{\begin{array}{l} d_{ij}exp\left(-\frac{{\left(\sin\theta_{ij}\left(t\right)-1\right)}^{2}}{2\sigma^{2}}\right),if\;\theta_{ij}\left(t\right)\leq 90^{{}^{\circ}}\\ d_{ij},\;\;\;\;\;\;\;\;\;\;\;\;\;\;\;\;\;\;\;\;\;\;\;\;\;\;\;\;\;\;\;\;\;\;\;\;if\;90^{{}^{\circ}}<\theta_{ij}\left(t\right)\leq 180^{{}^{\circ}} \end{array}$$

where $d_{ij}$ is the geographical distance between sites i and j, which can be calculated by their locations; $\theta_{ij}\left(t\right)$ is defined as the above, and it ranges in $\left[0,180^{{}^{\circ}}\right]$; $\theta_{ij}\left(t\right)$ and $d_{ij\_wind}\left(t\right)$ are the temporal variables, which can be computed by the dynamic real-time wind direction, because wind direction changes over time; σ is a tradeoff between the effect of geographical distance and wind direction. The dynamic wind field distance of upwind sites as defined above is smaller than their geographical distance. Accordingly, the evaluated affect degree increases. Meanwhile, the dynamic wind field distance of downwind sites remains equal to their geographical distance. The value of σ should be moderate. For example, Figure 4b shows the variation curve between $d_{ij\_wind}\left(t\right)$ and $\theta_{ij}\left(t\right)$ when $d_{ij}=1$ and σ=1,2,3. When σ=1, the wind field distance of the exact upwind location ($\theta_{ij}\left(t\right)=0)$ is 0.6 times its actual distance, thereby exaggerating the wind field effect. However, when σ increases to 3, the wind field distance of the exact upwind location is 0.95 times its actual distance; obviously, wind direction might not be functional in this situation. Thus, in our experiments, σ was set to 2. 

At the bottom of Figure 3 is an illustrated process of KNN-DWFD. In the case of predicting the PM_2.5_ concentration of the target station i at time T+1,…, T+24, we computed the dynamic wind field distance of all other sites at time T $d_{ij\_wind}\left(T\right)$. We selected and defined the K-nearest sites as neighbor sites. The historical air quality and meteorological data of the target and neighbor stations at r time points before time T+1 were chosen. Consequently, K+1 r∗f 2D matrices with time series were obtained (see the blue frames at the bottom of Figure 3), where K is the number of neighbor sites selected, and r represents the time lag. For example, when we use the historical data of the past 3 h to predict the future PM_2.5_ concentration, r refers to 3. f represents the number of features in consideration, and in our study, the features included air quality (PM_2.5_, PM_10_, NO_2_, CO, SO_2,_ and O_3_) and meteorological factors (weather, temperature, pressure, humidity, wind speed, and wind direction). Compared with the traditional KNN based on spatial distance, KNN-DWFD considers the effect of wind on the spreading of air pollutants. Therefore, it chooses more related sites than KNN based on geographical distance.

#### 2.2.2. LSTM-CNN: SRE

To perform a full excavation of the spatiotemporal correlations from the above K+1 r∗f 2D matrices, we constructed an SRE (see the *Spatiotemporal Relation Extractor* in Figure 3) that combined the LSTM characteristics that learns long- and short-term tendencies of input time series data along with the CNN characteristics that extracts and compresses spatiotemporal features from input data. The concrete flow of SRE is provided below. 

**Figure 5:**
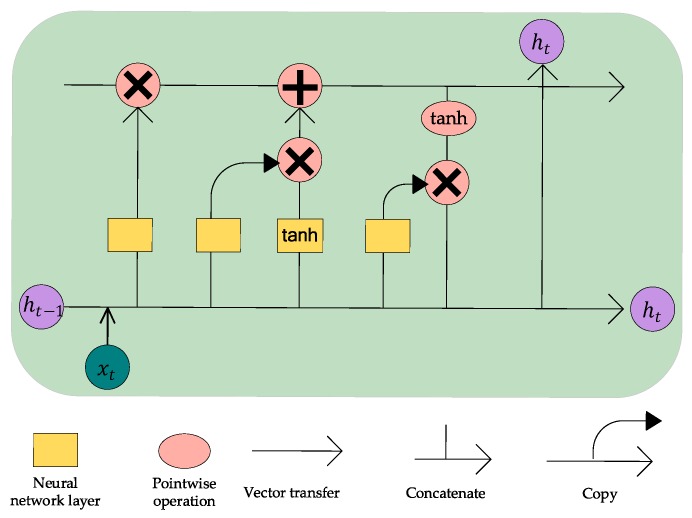
Structure of the recurrent memory cell of LSTM. $x_{t}$ and $h_{t}$ are the inputs and outputs of the memory cell, respectively.

First, LSTM was employed for conducting time series analysis. LSTM is a special recurrent neural network, with its recurrent neuron simultaneously learning long- and short-term tendencies of the time series data. The LSTM model used in our model was stateful LSTM, which uses the state of the current batch of LSTM samples as the initial state of the next batch of samples. It is more suitable for processing long-term time series data than the other models. As illustrated in the middle of Figure 3, we constructed local stateful LSTM models in the target and neighbor stations to capture long- and short-term tendencies from the historical air quality and meteorological data. Figure 5 shows the structure of the recurrent memory cell of LSTM, where $x_{t}$ represents the input, that is, the historical air quality and meteorological data of each site, and $h_{t}$ represents the output. The training process of the recurrent memory cell is given by the following equations.

Input gate: Decide what new information to store in the unit state, the information originated from the new observation at time t and the output of last time t−1:

(4) $$i_{t}=\sigma \left(W_{i}\cdot \left[h_{t-1},x_{t}\right]+b_{i}\right)$$

Forget gate: Selectively forget some past trends and other factors:

(5) $$f_{t}=\sigma \left(W_{f}\cdot \left[h_{t-1},x_{t}\right]+b_{f}\right)$$

Output gate: Determine the output information for the current observation:

(6) $$\tilde{C_{t}}=tanh\left(W_{C}\cdot \left[h_{t-1},x_{t}\right]+b_{C}\right)$$

(7) $$C_{t}=f_{t}*C_{t-1}+i_{t}*\tilde{C_{t}}$$

(8) $$o_{t}=\;\sigma \left(W_{o}\cdot \left[h_{t-1},x_{t}\right]+b_{o}\right)$$

(9) $$h_{t}=o_{t}*tanh\left(C_{t}\right)$$

where σ and tanh are activation functions; W and b are weight matrix and bias vector, respectively.

Second, a spatiotemporal matrix was constructed. The outputs of LSTM were input to a higher layer, namely FC layer, to output step temporal features. Here, step represents the dimension of predictions; it equals to 6 for 1–6 h prediction but equals to 2 for 7–12 and 13–24 h predictions. The temporal features were obtained via LSTM and FC. Hence, they were inherently correlated. We merged these temporal features of different sites into a 2D matrix (see the green matrix in Figure 3), in which each row represented a station’s temporal features extracted by local LSTM and FC. Accordingly, the adjacent elements of this matrix were correlated. The matrix is a spatiotemporal matrix, thereby forming the input of the next neural network.

**Figure 6:**
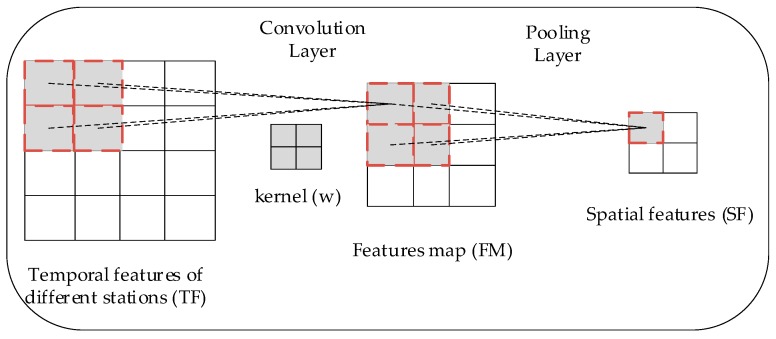
Training process of two-dimensional (2D)-CNN.

Finally, CNN was employed for further spatiotemporal analysis. CNN can automatically learn and detect the spatiotemporal features from the input high-dimensional matrix by the convolution layer. Thus, a 2D-convolution layer was used to extract spatiotemporal correlation from the above spatiotemporal matrix, and a pooling layer was used to conduct compression and output the spatiotemporal features. Figure 6 illustrates the process of CNN.

The left matrix TF in Figure 6 represents the spatiotemporal matrix obtained above. Kernel matrix is denoted by w, and the output feature map is denoted by FM. The elements of FM was obtained by the following equation:

(10) $$FM^{i,j}=\;g\left(\overset{P}{\underset{p=1}{\sum }}\overset{Q}{\underset{q=1}{\sum }}TF^{p+i-1,q+j-1)}w^{p,q}\right)$$

where g(·) is the activation function, and it was chosen as linear function. Superscript represents the element value of the corresponding position, and P as well as Q are the sizes of the kernels. To capture additional spatiotemporal features from TF, the number of kernels can be increased, and the number of obtained feature map can be increased accordingly. Then, the feature map FM was input into a pooling layer. The maximums of each submatrix with a pre-defined size (e.g., 2×2) was calculated, and they formed the final compressed spatiotemporal features SF. CNN can directly handle 2D spatiotemporal matrix without any flattening by convolution kernels and weight sharing. Compared with FC, CNN reduces the complexity of feature extraction, and additional deep spatiotemporal features can be extracted owing to the above advantages. 

#### 2.2.3. LSTM-CNN: WFC

Apart from past air quality and weather, the future weather condition is also highly related to the future PM_2.5_ concentration. To promote prediction accuracy, we integrated the weather forecasts, which included weather, wind direction, wind level, and up and bottom temperature, of the target station with the spatiotemporal features of past data extracted by SRE into FC. At this time, we output the PM_2.5_ predictions of the target site at the prediction time (See the pink frame at the top of Figure 3).

### 2.3. Evaluation Methods

The predictions of the next 24 h were divided into three intervals: 1–6, 7–12, and 13–24 h. As mentioned above, we predicted the real-valued concentration of PM_2.5_ for 1–6 h. Meanwhile, for 7–12 and 13–24 h, the maximum and minimum of PM_2.5_ concentration were forecasted, respectively. The evaluation criteria were the root mean square error (RMSE), the mean absolute error (MAE), and R-square ($R^{2}$). RMSE and MAE were employed to evaluate the prediction error of different models. Smaller values result in better prediction accuracy of the model. $R^{2}$ represents the fitting degree to the true PM_2.5_ observations of different models. A high value increases the reliability of the model. The formulas of these criteria are defined as follows:

(11) $$\mathrm{RMSE}=\sqrt{\frac{1}{n}\sum_{i=1}^{n}{\left(y_{i}-y_{i}^{*}\right)}^{2}},$$

(12) $$\mathrm{MAE}=\frac{1}{n}\sum_{i=1}^{n}\left|y_{i}-y_{i}^{*}\right|,$$

(13) $$R^{2}=\left(\frac{Cov\left(Y,\;Y^{*}\right)}{\sqrt{Var\left(Y\right)}\sqrt{Var\left(Y^{*}\right)}}\right){}^{2}\times 100\%,$$

where n is the sample size; $y_{i}$ and $y_{i}^{*}$ represent the observations and predictions of PM_2.5_, respectively; Cov(·) represents the covariance; Var(·) represents the variance; Y and $Y^{*}$ represent the predicted and observed sequences, respectively. Particularly, for the prediction task of the next 7–12 and 13–24 h, $y_{i}$ and $y_{i}^{*}$ represent the mean of the observed and predicted maximum and minimum PM_2.5_ concentration of each interval, respectively, and Y and $Y^{*}$ are the corresponding sequences of $y_{i}$ and $y_{i}^{*}$.

## 3. Results

The experimental data included the hourly air pollutant observations, meteorological factor observations, and weather forecasts of the 36 stations in Beijing from 1 May 2014 to 30 April 2015. A total of 8760 samples were collected for each station, and Section 2.1 introduces the detailed description of the data. Considering that evaluation results based on a single forecast origin can be unreliable when the forecasting results are sensitive to randomness and systematic business cycle effects [36], rolling origin has become a widely used evaluation technique in time series studies [22,37,38]. In the rolling origin method, the time series data are divided into several periods. The first several periods are selected as train set, and the next period is selected as test set. Then, the forecasting origin moves to the next period in turn and the forecasts are produced from each origin [39]. Rolling origin method partially controls for specific effects arising from a particular origin. In this study, considering the required sample size, the moving window and forecast window was set as one month. Considering the requirement of sample size, six-fold rolling origin experiments were conducted on each station in Beijing, and the results of totally 216 experiments were used to evaluate the performance of the proposed model. Table 1 shows the concrete time span of each train set and test set. It is worth noting that the train and test sets of each fold were determined in accordance with time sequence, guaranteeing they have no overlap.

Before building the LSTM-CNN-DWFD model, several parameters should be preset. According to Section 2.2.1, σ in the definition of dynamic wind field distance being 3 or 1 causes the wind impact to be considered inadequate or excessive. Therefore, we set σ equals to 2 in the following experiments. The best values of other parameters were determined via the rolling origin experiments on Site 1, where the number of neighbor sites selected K was set to 9, and the time lags r was set to 3, 6, and 12 for 1–6, 7–12, and 13–24 h, respectively. To avoid overfitting of the neural network, dropout layer and early stopping method were employed in our experiments. 

To demonstrate the effectiveness of the proposed LSTM-CNN-DWFD model, Section 3.1 presents the spatiotemporal distribution of its prediction error, from which we can obtain details of its prediction performance. In Section 3.2 and Section 3.3, we design a series of comparisons with six other methods to show its advantages in extracting spatiotemporal features and to confirm the superiority of the proposed KNN-DWFD method in selecting related sites. 

### 3.1. Performance of LSTM-CNN-DWFD Model

**Figure 7:**
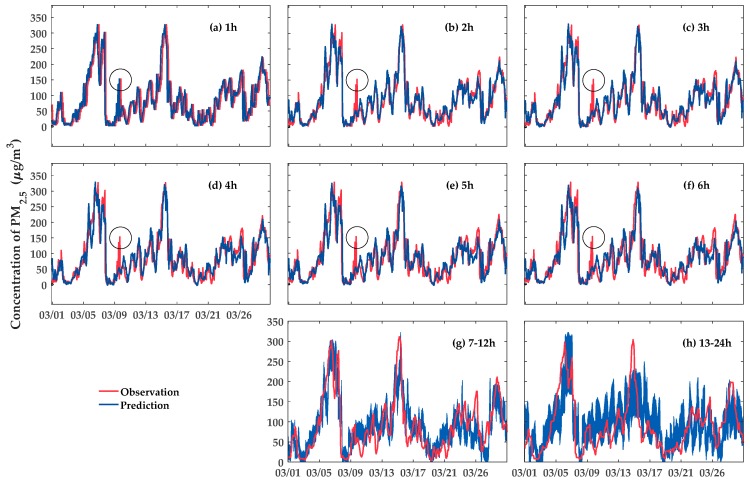
PM_2.5_ concentration observations versus predictions of Site 1 from 1 March 2015 to 31 March 2015. (**a**)–**f**) represent the comparison between the observations and predicted values for the next 1–6 h, respectively. (**g**) and (**h**) represent the comparison between the observations and predicted ranges for the next 7–12 and 13–24 h, respectively.

This subsection shows the forecasting performance of LSTM-CNN-DWFD model. Figure 7 intuitively shows the predicted PM_2.5_ concentration against the observations of Site 1 from 1 March 2015 to 31 March 2015. The red and blue lines represent the observations and forecasts of PM_2.5_ concentration, respectively. The $R^{2}$ values of the next 1–6 h prediction were 0.85, 0.81, 0.76, 0.70, 0.64, and 0.59, respectively. Accordingly, the explained variance of our model decreases as prediction time increases. As shown in Figure 7a–f, as prediction time increases, the prediction value of the peak value around 9 March (the circles in Figure 7) slowly decreased than the true value. Hence, a low prediction accuracy occurs. Additionally, (g) and (h) in Figure 7 show that, during 13–24 h, the range of the predicted PM_2.5_ concentration was larger than that in 7–12 h. In other words, the level of uncertainty increases in 13–24 h. However, the mean of the observations of each interval (red lines) constantly fell in the predicted range. 

Table 2 shows each fold’s prediction error of LSTM-CNN-DWFD model. The lowest RMSE and MAE of all folds occurred at the next 1–6 h, followed by the values at 7–12 h. By contrast, the values at 13–24 h were typically the highest. Thus, the prediction accuracy tends to decrease as the prediction time increases. The average RMSE (MAE) of 216 experiments were 43.90 (29.17), 57.89 (42.16), and 63.14 (47.64) for 1–6, 7–12, and 13–24 h, respectively. The standard errors of RMSE (MAE) of 36 stations were 9.48 (6.84), 12.13 (9.16), and 12.73 (9.88), respectively, which illustrates the stability of LSTM-CNN-DWFD model. Additionally, the RMSEs of the 1–6, 7–12, and 13–24 h predictions in Fold1–4 ranged in 45–52, 58–73, and 62–82, respectively. However, in Fold5–6, the RMSEs ranged in 34–35, 42–47, and 44–52, respectively. Hence, the RMSEs of Fold5–6 were relatively lower in general than those in other folds. The results of MAE also show the same trend. Therefore, the prediction accuracy in spring (Fold5–6) tends to be higher than in autumn and winter (Fold1–4).

**Figure 8:**
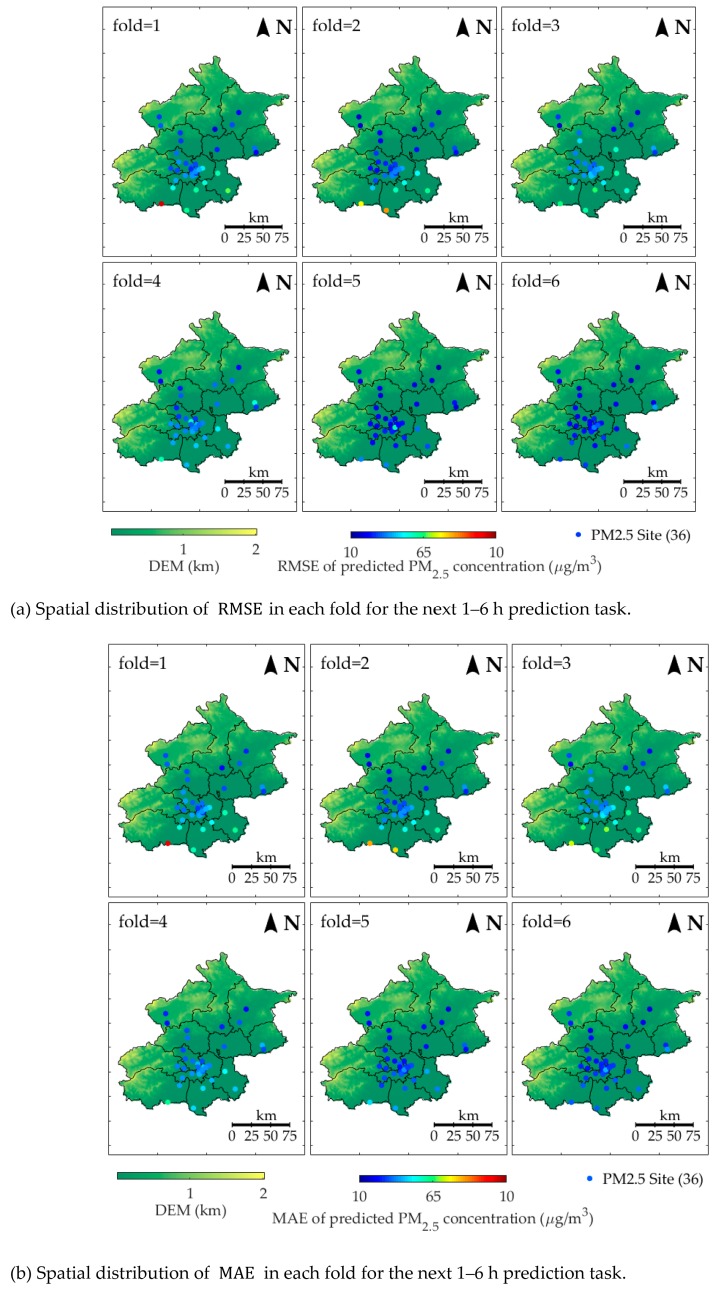
Spatial distribution of prediction error of LSTM-CNN-DWFD in each fold for the next 1–6 h prediction task.

Figure 8 presents the spatial distribution of prediction error in each fold for the next 1–6 h prediction task. The bluer the color, the lower the prediction error (RMSE or MAE). 

As shown in Figure 8, the color of sites becomes bluer as fold increases. Thus, the prediction error decreases. The spatial distribution of the prediction error of all folds shows that the RMSE and MAE of southern sites were higher than those of northern sites. Hence, the prediction performance in northern sites are better than that in southern sites.

### 3.2. Effectiveness of LSTM-CNN-DWFD in Extracting Spatiotemporal Correlation 

We proposed a multi-step-ahead forecasting model LSTM-CNN-DWFD in this paper. The LSTM-CNN part was designed to extract spatiotemporal correlation from the input data. To show the effectiveness of its architecture, we conducted three groups of comparison experiments between LSTM-CNN and five baseline models. We chose the neighbor sites in accordance with geographical distance in all models.

(a) Evaluate the effect of neighbor sites’ data: LSTM-NN versus LSTM-CNN, CNN, and LSTM-FC [22]. LSTM-NN adopted an LSTM layer to capture the temporal trend of the historical data of target station and used FC layer to integrate weather forecasts. It did not consider the spatiotemporal data of neighbor sites. However, CNN, LSTM-FC, and LSTM-CNN included the historical data of target and neighbor stations as well as weather forecasts as input.

(b) Evaluate the effectiveness in feature extraction: LSTM-CNN versus CNN and LSTM-FC. The input data of these models were the same, but their neural network differed. CNN adopted a convolution layer and a pooling layer to capture spatiotemporal features and integrated weather forecasts by an FC layer. The LSTM-FC separately trained local LSTM models in the target and neighbor sites similar to LSTM-CNN model. However, it adopted FC to extract the spatial feature from the outputs of local LSTM models. Finally, the weather forecasts were also integrated by FC layer in LSTM-FC. 

(c) Evaluate the effect of weather forecasts: LSTM-NN versus LSTM and APNet [27]. LSTM and APNet considered the historical data of the target station as input, thereby neglecting the effect of weather forecasts. LSTM used an LSTM layer to capture temporal correlation of the input. APNet first used three 1D-convolution and batch normalization layers to compress the input data; it then used LSTM to capture temporal features.

For fairness, all of the LSTM layers used above were stateful LSTM. These models were trained and tested on 36 stations using six-fold rolling origin, and the mean RMSE, MAE, and $R^{2}$ of the total 216 experiments can provide us their general prediction performance.

Table 3 clearly shows the prediction error of different methods in three different prediction intervals. The best prediction performance (the smallest RMSE and MAE and the largest $R^{2}$) of each column is marked in boldface. As shown in Table 3, LSTM-NN showed higher RMSE and MAE and lower $R^{2}$ at all prediction scales than CNN, LSTM-FC, and LSTM-CNN. This result is explained by the LSTM-NN neglecting the spatiotemporal correlation of neighbor sites, thereby causing its low prediction accuracy. The RMSEs of the proposed LSTM-CNN model for 1–6, 7–12, and 13–24 h were 44.68, 58.77, and 63.40, respectively, and all RMSEs were lower than those of CNN and LSTM-FC. From the aspect of MAE and $R^{2}$, LSTM-CNN performed the best in the 1–6 and 13–24 h prediction tasks with lower MAE and higher $R^{2}$. Therefore, in 1–6 and 13–24 h predictions, LSTM-CNN has the highest prediction accuracy. In 7–12 h prediction task, CNN, LSTM-FC, and LSTM-CNN, respectively showed the best performance in accordance with $R^{2}$, MAE, and RMSE. Therefore, the overall performance of the three models can be regarded as close in 7–12 h predictions. In addition, the RMSE and MAE of APNet and LSTM were obviously higher than those of LSTM-NN, where their $R^{2}$ value were much lower. Hence, neglecting weather forecasts causes substantial loss to the prediction accuracy.

### 3.3. Effectiveness of LSTM-CNN-DWFD in Selecting Related Sites

The KNN-DWFD part in the LSTM-CNN-DWFD model was designed to select K neighbor sites considering wind impact. This subsection compares the prediction performance of the forecasting model under KNN in accordance with geographical distance (in LSTM-CNN model) and dynamic wind field distance (in LSTM-CNN-DWFD model).

#### 3.3.1. The Difference of KNN and KNN-DWFD in Selecting Neighbor Sites

**Figure 9:**
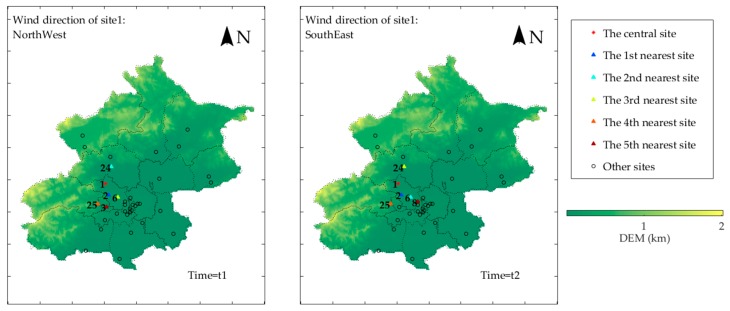
Illustration of the five nearest stations to Site 1 under different wind directions according to dynamic wind field distance.

To demonstrate the process of using KNN-DWFD for selecting neighbor sites, we compared the five nearest sites to Site 1 under different wind directions in accordance with dynamic wind field distance in Figure 9. Here, * represents Site 1, and the triangles represent the five nearest sites. The bluer the color, the nearer the site, and the higher its affect degree.

As shown in Figure 9, Sites 6 and 24 are approximately the same geographical distance from Site 1; however, they are at different directions, thereby causing their ranks of affect degree to change with wind direction. At time $t_{1}$ when the wind direction of Site 1 was northwest, Site 24 was in the upwind area, thereby making it more related to Site 1 than the other sites (excluding the nearest surrounding site Site 2). By contrast, at time $t_{2}$, when the wind direction of Site 1 changed to southeast, Site 6 was nearly in the exact upwind area. Hence, the affect degree of Site 6 was higher than that of other sites (excluding the nearest surrounding site Site 2). This illustrates that KNN-DWFD can dynamically select the most related surrounding sites according to dynamic wind field.

#### 3.3.2. Comparing Prediction Performance of LSTM-CNN and LSTM-CNN-DWFD

Six-fold rolling origin experiments were performed to build LSTM-CNN and LSTM-CNN-DWFD models in 36 stations. Table 4 shows the mean RMSE, MAE, and $R^{2}$ at multiple prediction scales, where Models 1 and 2 represent LSTM-CNN and LSTM-CNN-DWFD, respectively. In 1–6 and 7–12 h prediction tasks, LSTM-CNN-DWFD showed low RMSE and MAE and high $R^{2}$, thereby indicating a high prediction accuracy. For 13–24 h prediction task, the MAE of LSTM-CNN-DWFD is 47.54, which was slightly higher than that of LSTM-CNN. However, the RMSE of LSTM-CNN-DWFD was 63.14, and $R^{2}$ was 34.21%, both of which showed better performance than those of LSTM-CNN. Thus, LSTM-CNN-DWFD performed higher accuracy than LSTM-CNN in all prediction scales. In 7–12 h prediction, LSTM-CNN, LSTM-FC, and CNN respectively had the lowest RMSE, the lowest MAE, and the highest $R^{2}$ as shown in Table 3. However, LSTM-CNN-DWFD further improved the prediction accuracy of LSTM-CNN from all aspects. The RMSE, MAE, and $R^{2}$ of LSTM-CNN-DWFD for 7–12 h prediction were 57.89, 42.16 and 49.43%, respectively, all of which outperformed those of LSTM-CNN, LSTM-FC, and CNN. Therefore, from all kinds of criteria, such as RMSE, MAE, and $R^{2}$, LSTM-CNN-DWFD has the best prediction performance.

The density of stations highly affects the significance of spatial correlations. The higher the density, the nearer neighbor sites, and the more significant the spatial correlation. Table 5, Table 6, and Table 7 show the prediction error of LSTM-CNN and LSTM-CNN-DWFD in regions with different densities of stations at three different prediction intervals. Here, #ns is the number of surrounding sites within 1.5 km to the target station, which represents the density of stations. The number in the bracket stands for the number of target stations that locate the corresponding density area.

The distribution of stations in Beijing is uneven. A total of 15 stations have no more than two sites within 1.5 km. Meanwhile, nine sites have more than 12 sites within the same distance range. The comparison among Table 5, Table 6, and Table 7 shows that the highest prediction accuracy for 1–6, 7–12, and 13–24 h prediction tasks of both models all occurred in 2<#ns≤12 region, as a lower RMSE and MAE and a higher $R^{2}$ indicated. However, the accuracy in #ns≤2 and #ns>12 regions was relatively worse.

Nonetheless, for #ns≤2, the RMSEs of LSTM-CNN-DWFD at 7–12 and 13–24 h are 62.35 and 67.24, respectively, both of which were lower than that of LSTM-CNN. For 2<#ns≤12 and #ns>12 region, the RMSE and MAE of LSTM-CNN-DWFD were generally all lower than LSTM-CNN at multiple prediction scales (except for the MAE at 13–24 h prediction in 2<#ns≤12 region), and $R^{2}$ of LSTM-CNN-DWFD were all higher. Hence, LSTM-CNN-DWFD showed a better prediction accuracy at all prediction scales and all regions with different densities than the other models. In addition, as the density of stations increases, the difference among the RMSE, MAE, and $R^{2}$ of the two models increases, which means the superiority of LSTM-CNN-DWFD increases in areas where spatial correlation is important.

## 4. Discussion

**Figure 10:**
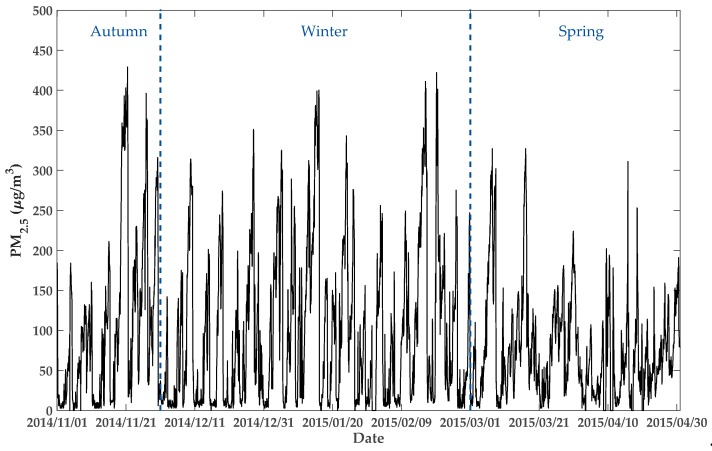
Variation curve of hourly observations of PM_2.5_ concentration of Site 1 from 1 Nov 2014 to 30 Apr 2015 (i.e., the time span of all test sets).

This paper proposed a novel PM_2.5_ forecasting model―LSTM-CNN-DWFD, which constructed a hybrid neural network to extract spatiotemporal data, and took wind impact into consideration when selecting related surrounding sites. To demonstrate the advantage of the proposed model, six-fold rolling origin experiments were conducted, and Section 3 shows the results. The experimental data was restricted to a single year, and the test sets covered part autumn (Nov 2014 in Fold1), a whole winter (Dec 2014 to Feb 2015 in Fold 2–4), and part spring (Mar 2015 to Apr 2015 in Fold 5–6). As shown in Table 2 and Figure 8, from the view of season, LSTM-CNN-DWFD performed better in spring than in autumn and winter; from the view of space, LSTM-CNN-DWFD performed better in the north than in the south. Similar results were obtained by Zhao [22] and Bai [40], both of which found that the performance in winter was the worst, followed by autumn, spring, and summer. Thus, the proposed model is expected to perform higher accuracy if the test set is expanded to a longer time period. The seasonal difference in the prediction accuracy resulted from the variations of atmospheric environment and human activities. The atmosphere environment in autumn and winter (Nov 2014 to Feb 2015) was more stable than spring (Mar 2015 to Apr 2015), including a lower temperature (0.96 versus 10.72 ℃) and lower wind speed (7.16 versus 7.72 m/s). The stable atmosphere structure in winter contributed weak diffusion of PM_2.5_ in both horizontal and vertical directions. In addition, human activities in winter (e.g., heating and use of festival firecrackers) contributed anthropogenic emission. According to Liang and Zou [41], heating activities in winter has contributed more than 50% increase (on average) in PM_2.5_ concentration in Beijing since 2010. Ye and Chen [42] found that the traditions of exploding firecrackers had a direct effect on the air pollution aggravation during the Chinese New Year. As a result, the variations of PM_2.5_ concentration in winter were more dramatic and the peaks were higher, as Figure 10 shows. Consequently, higher contribution of anthropogenic emission in winter and higher peaks of PM_2.5_ concentration caused predicting air quality based on meteorological factors more difficult, and the prediction accuracy was lower. The spatial difference was caused due to that the pollution condition is worse in the south (as shown in Figure 2), and the monitoring stations in the south are fewer and farther between than those in north. Hence, the CNN-based spatial relation extractor cannot capture the spatial dependence well.

From the comparison results between LSTM-CNN and five baseline models in Table 3, three useful findings can be extracted.

(1) From comparison (a), CNN, LSTM-FC, and LSTM-CNN exhibited lower RMSE and MAE and higher $R^{2}$ than those in LSTM-NN. This result is explained by neighbor stations having a high effect on the pollution of the target station due to the transport of pollutants. Similar conclusions were also drawn by Zhao [22] and Wen [28] by comparing the performance of the models with and without the surrounding sites considered. Therefore, considering related neighbor stations can further improve prediction accuracy.

(2) From comparison (b), LSTM-CNN had higher prediction accuracy, especially in 1–6 and 13–24 h, than those of CNN and LSTM-FC. This result is due to the special architecture of combining LSTM with CNN in the SRE part of LSTM-CNN. Compared with CNN, the LSTM layer in SRE is more suitable for processing time series data. The recurrent cell of LSTM contains input gate, forget gate, and output gate. The three gates make the recurrent neuron able to store long-term tendency of the input time series data and extract useful short-term tendency at the same time. This was also confirmed in the work of Li and Peng [11]. However, CNN do not have a recurrent neuron in its architecture, therefore it cannot learn the temporal dependency of time series data. Hence, LSTM can more efficiently extract temporal features than CNN. By comparing the performance of air quality prediction on city scale of CNN-alone and LSTM-alone models, Qin and Yu [26] also found that CNN performance was poor in dealing with long-term sequence prediction. In addition, compared with LSTM-FC, the CNN layer in SRE can directly handle the 2D spatiotemporal matrix and extract spatiotemporal features therefrom. However, FC can only employ 1D data as input, so 2D spatiotemporal matrix must be flattened to 1D data to be processed by FC. The flattening process causes some loss of the spatiotemporal dependency among the element of 2D matrix. Therefore, the spatiotemporal information can be more fully utilized by CNN than FC, and additional deep spatiotemporal features can be extracted. By combining LSTM with CNN in SRE, the proposed LSTM-CNN model showed higher prediction accuracy than CNN and LSTM-FC.

(3) From comparison (c), LSTM-NN performed better than LSTM and APNet, especially at 13–24 h. This illustrates that weather forecast data are highly related to the future PM_2.5_ concentration, especially for long-term prediction. Introducing the weather forecasts can improve prediction performance.

Compared with LSTM-CNN, which selected neighbor sites in accordance with geographical distance, LSTM-CNN-DWFD selected neighbor sites in accordance with dynamic wind field distance, and obtained higher prediction accuracy. Table 4–7 provide the comparison of the prediction accuracy between LSTM-CNN and LSTM-CNN-DWFD. On the basis of the results, both models performed worse in #ns≤2 and #ns>12 regions. The bad performance, namely, the low prediction accuracy, results from the number of neighbor stations K set as 9 in our experiments. For #ns≤2, some less relevant sites were introduced into the model. Meanwhile, for #ns>12, some high relevant sites were ignored in the model. Therefore, a more adaptive selection method can be explored to make the number of selected surrounding sites be able to be adaptive to different density of sites. Nonetheless, the results show that LSTM-CNN-DWFD performed well for 1–6, 7–12, and 13–24 h prediction tasks and all regions with different densities. Moreover, the higher the density of stations, the more important the spatial correlation, and the more significant the superiority of LSTM-CNN-DWFD. This result is explained by spatial correlation being anisotropy which is affected by wind. However, geographical distance describes that the spatial dependency is affected by distance, and it takes spatial correlation to be isotropy. In this study, the proposed dynamic wind field distance introduced wind direction into the evaluation of the distance between sites, making it more suitable to represent the spatial relations between sites than geographical distance. Consequently, the neighbor sites selected in LSTM-CNN-DWFD contributed more to the PM_2.5_ concentration prediction of target station than the neighbor sites selected in LSTM-CNN. Similar trends also occurred in some studies on spatial interpolations of air pollutants. By introducing wind direction and wind speed into the evaluation of the distance between sites, Li [43] and Li [44] both improved the accuracy of spatial interpolation of pollutants. However, both of them did not discuss the applications in air quality forecasting. Therefore, in the future study, wind speed can be introduced to the definition of wind field distance, and we believe that the prediction performance of the forecasting model will be better.

Compared with six other methods, the proposed LSTM-CNN-DWFD model showed the highest prediction accuracy in forecasting hourly PM_2.5_ concentration. The LSTM-CNN architecture is shown to be more effective in extracting spatiotemporal features, and dynamic wind field distance fits the spatial correlation better than geographical distance. Due to the limited sample size of the employed dataset, the performance of summer and autumn was not evaluated enough. However, as mentioned above, many related studies demonstrated that the performance of summer and autumn were better than winter [22,40]; thus, the proposed model is believed to have a higher accuracy if a longer time period is covered in the test set.

## 5. Conclusions

This study presented a site-specific forecasting model, namely, LSTM-CNN-DWFD, to predict air pollutant concentrations over the next 24 h using historical air quality, meteorological data, and weather forecasts. By combining LSTM and 2D-CNN, the proposed model simultaneously handled long- and short-term temporal trends and spatial dependency of the spatiotemporal data. Additionally, using a new KNN method, namely, KNN-DWFD, highly related neighbor stations were chosen in the model with wind effect considered. Finally, accurate and stable predictions were realized via the combination of KNN-DWFD and LSTM-CNN in LSTM-CNN-DWFD. Furthermore, through the six-fold rolling origin comparison experiments for 1–6, 7–12, and 13–24 h prediction tasks conducted on the 36 stations in Beijing, LSTM-CNN-DWFD has the highest prediction accuracy, taking RMSE, MAE, and $R^{2}$ as indicators. The following are the main findings of this study:

1. The historical air quality and meteorological data of neighbor stations are valuable spatiotemporal data, and fully utilizing these data can considerably improve prediction accuracy. Additionally, taking weather forecasts into consideration can also help predict the future PM_2.5_ concentration, especially for long-term prediction.
2. The proposed model, namely, LSTM-CNN, can more efficiently capture the spatiotemporal features by combining local LSTM models and CNN than CNN and LSTM-FC. Hence, it exhibited better prediction performance than the other models as indicated by its low RMSE and MAE and high $R^{2}$.
3. We proposed a dynamic wind field distance to replace geographical distance in new KNN method—KNN-DWFD. The comparison results show that it can fit the spatial correlation better than geographical distance. LSTM-CNN-DWFD is more capable of adapting to different prediction time and density levels than LSTM-CNN, thereby providing more accurate and stable predictions as indicated by its low RMSE and MAE and high $R^{2}$.

Future studies should focus on the following aspects: (1) Develop a method to choose the number of neighbor stations adaptively for areas with different densities of stations so that the forecasting model can fit the spatial correlations well accordingly; (2) explore a wind field distance definition that simultaneously considers the impact of wind speed and direction and not only the wind direction; (3) explore other patterns to introduce wind impact into the spatial dependency.

## Figures and Tables

**Table 1:** Time span of six-fold rolling origin.

Fold	Train	Test
Fold1	2014.05–2014.10	2014.11
Fold2	2014.05–2014.11	2014.12
Fold3	2014.05–2014.12	2015.01
Fold4	2014.05–2015.01	2015.02
Fold5	2014.05–2015.02	2015.03
Fold6	2014.05–2015.03	2015.04

**Table 2:** Each fold’s prediction error of LSTM-CNN-DWFD model.

Fold	1–6 h		7–12 h		13–24 h	
	RMSE	MAE	RMSE	MAE	RMSE	MAE
Fold1	50.13	32.73	72.42	50.86	81.51	59.38
Fold2	45.26	29.79	58.87	43.76	62.61	48.58
Fold3	51.41	34.70	65.81	48.17	69.21	53.40
Fold4	46.93	30.14	61.83	44.43	69.61	51.88
Fold5	34.91	24.34	46.14	33.92	51.25	38.65
Fold6	34.74	23.33	42.28	31.79	44.65	33.97
Mean	43.90	29.17	57.89	42.16	63.14	47.64

**Table 3:** Comparison of the prediction error of six models in three forecast intervals.

Model	1–6 h			7–12 h			13–24 h		
	RMSE	MAE	$R^{2}\;\left(\%\right)$	RMSE	MAE	$R^{2}\;\left(\%\right)$	RMSE	MAE	$R^{2}\;\left(\%\right)$
APNet	52.71	35.72	62.72	67.10	49.34	32.07	72.08	55.14	15.34
LSTM	47.03	30.69	68.23	64.12	46.22	37.76	69.10	52.44	20.59
LSTM-NN	46.18	30.40	69.87	60.49	44.04	45.72	63.94	48.47	33.39
CNN	45.10	29.76	71.48	59.27	42.77	**49.05**	63.85	48.26	33.76
LSTM-FC	45.29	30.04	70.65	59.10	**42.27**	47.82	63.53	47.71	33.63
LSTM-CNN	**44.68**	**29.60**	**71.71**	**58.77**	42.32	47.95	**63.40**	**47.54**	**33.78**

Note: The best prediction performance (the smallest RMSE and MAE and the largest $R^{2}$) of each column is marked in boldface.

**Table 4:** Comparison results of LSTM-CNN and LSTM-CNN-DWFD for all sites.

Model	1–6 h			7–12 h			13–24 h		
	RMSE	MAE	$R^{2}\;\left(\%\right)$	RMSE	MAE	$R^{2}\;\left(\%\right)$	RMSE	MAE	$R^{2}\;\left(\%\right)$
Model 1	44.68	29.60	71.71	58.77	42.32	47.95	63.40	**47.54**	33.78
Model 2	**43.90**	**29.17**	**72.56**	**57.89**	**42.16**	**49.43**	**63.14**	47.64	**34.21**

Notes: Models 1 and 2 represent LSTM-CNN and LSTM-CNN-DWFD, respectively. The best prediction performance (the smallest RMSE and MAE and the largest $R^{2}$) of each column is marked in boldface.

**Table 5:** Comparison results of LSTM-CNN and LSTM-CNN-DWFD in #ns≤2 area (15 sites).

Model	1–6 h			7–12 h			13–24 h		
	RMSE	MAE	$R^{2}\;\left(\%\right)$	RMSE	MAE	$R^{2}\;\left(\%\right)$	RMSE	MAE	$R^{2}\;\left(\%\right)$
Model 1	**49.27**	**30.56**	**73.68**	62.40	**41.84**	54.64	67.45	46.74	42.15
Model 2	49.59	30.74	73.45	**62.35**	42.00	**54.85**	**67.25**	**46.71**	**42.50**

**Table 6:** Comparison results of LSTM-CNN and LSTM-CNN-DWFD in 2<#ns≤12 area (12 sites).

Model	1–6 h			7–12 h			13–24 h		
	RMSE	MAE	$R^{2}\;\left(\%\right)$	RMSE	MAE	$R^{2}\;\left(\%\right)$	RMSE	MAE	$R^{2}\;\left(\%\right)$
Model 1	44.82	28.96	74.61	59.12	41.31	52.52	63.86	**46.42**	37.67
Model 2	**43.48**	**28.28**	**75.81**	**58.09**	**41.14**	**54.06**	**63.59**	46.75	**38.12**

**Table 7:** Comparison results of LSTM-CNN and LSTM-CNN-DWFD in #ns>12 area (9 sites).

Model	1–6 h			7–12 h			13–24 h		
	RMSE	MAE	$R^{2}\;\left(\%\right)$	RMSE	MAE	$R^{2}\;\left(\%\right)$	RMSE	MAE	$R^{2}\;\left(\%\right)$
Model 1	46.24	29.45	72.79	63.98	45.19	45.07	70.12	51.50	25.65
Model 2	**44.13**	**28.23**	**74.84**	**62.38**	**44.57**	**47.36**	**68.94**	**51.33**	**28.11**

Notes: Models 1 and 2 represent LSTM-CNN and LSTM-CNN-DWFD, respectively. Here, #ns represents the number of surrounding sites within 1.5 km to the target station. The number in the brackets stands for the number of target stations that locate the corresponding density area. The best prediction performance (the smallest RMSE and MAE and the largest $R^{2}$) of each column is marked in boldface.
